# High-Intensity Interval Exercise Performance and Short-Term Metabolic Responses to Overnight-Fasted Acute-Partial Sleep Deprivation

**DOI:** 10.3390/ijerph18073655

**Published:** 2021-04-01

**Authors:** Zacharias Papadakis, Jeffrey S. Forsse, Andreas Stamatis

**Affiliations:** 1 Human Performance Laboratory, Barry University, Miami Shores, FL 33138, USA; 2 Baylor Laboratories for Exercise Science and Technologies, Baylor University, Waco, TX 40385, USA; jeff_forsse@baylor.edu; 3 SUNY Plattsburgh, Plattsburgh, NY 12901, USA; astam004@plattsburgh.edu

**Keywords:** sleep restriction, metabolism, cardiorespiratory fitness, PSQI, SenseWear

## Abstract

People practicing high-intensity interval exercise (HIIE) fasted during the morning hours under a lack of sleep. Such a habit may jeopardize the health benefits related to HIIE and adequate sleep. Fifteen habitually good sleeper males (age 31.1 ± 5.3 SD year) completed on a treadmill two isocaloric (500 kcal) HIIE sessions (3:2 min work:rest) averaged at 70% VO_2reserve_ after 9–9.5 h of reference sleep exercise (RSE) and after 3–3.5 h of acute-partial sleep deprivation exercise (SSE). Diet and sleep patterns were controlled both 1 week prior and 2 days leading up to RSE and SSE. HIIE related performance and substrate utilization data were obtained from the continuous analysis of respiratory gases. Data were analyzed using repeated measures ANOVA with the baseline maximum oxygen uptake (VO_2max_) and body fat percentage (BF%) as covariates at *p* < 0.05. No difference was observed in VO_2max_, time to complete the HIIE, VE, RER, CHO%, and FAT% utilization during the experimental conditions. Whether attaining an adequate amount of sleep or not, the fasted HIIE performance and metabolism were not affected. We propose to practice the fasted HIIE under adequate sleep to receive the pleiotropic beneficial effects of sleep to the human body.

## 1. Introduction

Sleep research is commonly focused on acute-total and chronic-partial deprivation [1,2] with sleep related parameters to be either self-assessed (e.g., Pittsburgh Sleep Quality Index–PSQI) [3,4,5,6,7,8,9,10] and/or objectively assessed with a sophisticated apparatus (e.g., sleep monitors, polysomnography, actigraphy) [4,5,6,9,10,11,12]. Few studies have examined the acute-partial sleep deprivation with respect to the exercise performance, with most of them focusing on anaerobic performance (e.g., Wingate). Results are conflicting, as some indicate that partial sleep deprivation negatively impacts the next day’s performance, especially the afternoon’s compared to next morning’s performance [13,14,15], while others reported no difference in Wingate performance after 4 h of partial sleep deprivation [13,14,16]. In addition, partial sleep deprivation at the end of the night decreases anaerobic performance compared to the deprived condition at the beginning of the night among Judokas [16]. The inconsistency in the aforementioned findings seems to be attributed to the differences in the examined population, applied exercise protocols, heterogeneity of the examined variables [17], and to combinations of sleep deprivation protocols in the form of delayed onset, intermittent waking, and early rising [6].

Even fewer studies have focused on acute-partial sleep deprivation on aerobic performance, also yielding contradictory findings. Antunes et al. reported that sleep quality and duration assessed by PSQI are associated with the maximal incremental cycle ergometer performance and appears to be fitness independent. The maximum heart rate (HR_max_) was positively correlated with sleep quality (r = 0.41, *p* = 0.03) and negatively correlated with sleep duration (r = − 0.43, *p* = 0.02). Good quality sleepers, as indicated by the PSQI scores, presented higher values of maximum oxygen consumption (VO_2max_) and lower HR_max_ compared to the individuals with altered sleep (i.e., <7 h) [3]. Partial sleep deprivation of 3 h sleep did not alter the intermittent aerobic performance (i.e., Yo-Yo) of Taekwondo athletes in the morning of the following day (7–8 a.m.) [18]. Similarly, about 3 h of deprived sleep did not significantly decrease the next day’s graded exercise performance on a cycle ergometer between 5 p.m. and 9 p.m. (i.e., peak VO_2max_ by 15%, peak heart rate by 1.6%, and exercise duration by 3.2%) compared to about 8 h of reference sleep [19]. Forty minutes of cycling at a self-selected pace (~64.4 ± 16% SD VO_2max_) with the goal to cover as much distance as possible did not alter the exercise performance after 4 h of sleep compared to the reference sleep [20]. Similarly, no difference was reported in the heart rate and energy expenditure after two 6-min early morning runs at 60% of running treadmill speed after 4 h of sleep compared to 7.5 h [21]. Mougin et al. reported no differences on the maximal sustained exercise intensity and a significant decrease in VO_2max_, ventilation, and heart rate during the incremental cycle ergometry performed at 2 p.m. in the next day after 3 h of partial sleep deprivation [22].

In contrast to reporting no change or mixed results in aerobic exercise performance, Mougin et al. found a decrease in the maximal work rate during incremental cycling to exhaustion after ~4 h of sleep in the evening of the next day [23]. The next morning’s 3-km time trial performance on a cycle ergometer was significantly impaired by 4% following partial sleep deprivation (~3 h) compared to a full night of sleep (~7 h), with no reported changes for maximum oxygen consumption, expired ventilation, and respiratory exchange ratio [6]. In comparison, it was shown that 4 h of sleep impairs the aerobic cycling performance on recreational cyclists by 4.1% compared to normal sleep (~8 h) [10]. Moreover, trained cyclists/triathletes also reported an impaired cycling time trial performance on a partial sleep deprivation (~4.7 h) condition compared to normal sleep (~7 h) [24].

Findings on partial sleep deprivation effects on aerobic related exercise performance are mixed. The reason for this discrepancy is attributed to the small sample size (e.g., less than 10 participants) and subsequent lack of power to extrapolate the reported results, differences in the selected populations, durations of sleep deprivation, and performance assessment protocols [18,25]. In this context, the majority of the reported literature has been focused on examining the link between sleep and athletic performance [3,6,9,25,26,27,28,29,30,31] and/or the link between diseases and their progression [1,32,33,34,35,36,37,38,39,40,41]. Lack of sleep is associated with several metabolic dysfunctions, influencing lifestyle factors such as nutritional balance/metabolism, body composition, and cardiorespiratory fitness (CRF), ultimately jeopardizing an individual’s health status [17,42,43,44].

Growing evidence favors high-intensity interval exercise (HIIE) for achieving various health benefits [45]. HIIE’s popularity is mainly based on the fact that compared to other forms of exercise, it can be completed in just 20–30 min per session [46]. Since lack of time is the most common excuse that people give for not exercising [47], the HIIE session may be a time-efficient strategy to improve health, body composition, and CRF levels [48]. People of varying CRF levels and body fat percentages (BF%) in their effort to accomplish everything in a 24/7 society may practice HIIE, early in the morning before they engage in their busy lives under the influence of lack of sleep [49,50]. It is possible for people that select to work out early in the morning to exercise after an overnight-fasted state (e.g., empty stomach) in an effort to improve fuel efficiency and utilization during exercise, as well as to increase fat usage in the premise to stimulate the accumulated weight loss [51,52,53,54]. Evidence suggests that exercise after an overnight fasting in healthy men can enhance training-induced adaptations in the muscle metabolic profile. Energy production during exercise in the overnight-fasted state is supported by both fat and carbohydrates utilization at 45–65% of VO_2max_ and predominantly from carbohydrates for intensities over 65% of VO_2max_ [52,55], while fat oxidation is increased during exercise in the overnight-fasted state [56,57].

However, such health-related behaviors may have detrimental effects on exercise performance and the related metabolic, oxidative, and inflammatory responses [9,42,58]. It is possible for people when they perform HIIE early in the morning under acute-partial sleep deprivation to experience decrements in physical performance (e.g., VO_2max,_) due to an increase in the metabolism (e.g., insulin resistance, glucose tolerance), inflammation (e.g., tumor necrosis factor alpha and prostaglandin E_2_), autonomic nervous system (sympathetic activity), perceived exertion, and impairment in the aerobic metabolic pathways [26,44,58,59] presenting an increase in carbohydrate (CHO) utilization due to an increase in ghrelin, hepatic glucose production [60], and increase in the respiratory exchange ratio (RER) [58].

The influence of acute-partial sleep deprivation on the HIIE performance and metabolism is less investigated and physiological parameters that may influence the HIIE performance and substrate utilization/partitioning are poorly understood [43]. Most of the sleep and exercise accumulated evidence on both anaerobic and aerobic performances is based on cycling ergometry protocols and evening performance assessments. It is possible for exercising on a treadmill in the morning after an overnight-fasted acute-partial sleep deprivation to elicit different performance responses and substrate utilization compared to the reference sleep and exercise. Therefore, it was hypothesized (a) that the HIIE aerobic performance (e.g., VO_2max_, time to expend 500 kcal, expired ventilation, and respiratory exchange ratio) of apparently healthy fit males will be impaired after 3–3.5 h of acute-partial-deprived sleep exercise (SSE) condition compared to 9–9.5 h of reference sleep HIIE (RSE) condition, and (b) that the carbohydrate percentage (CHO%) utilized during HIIE of apparently healthy fit males will be increased and the fat percentage (FAT%) utilized will be decreased in SSE compared to the RSE condition.

## 2. Materials and Methods

### 2.1. Study Design and Participants

**Figure 1:**
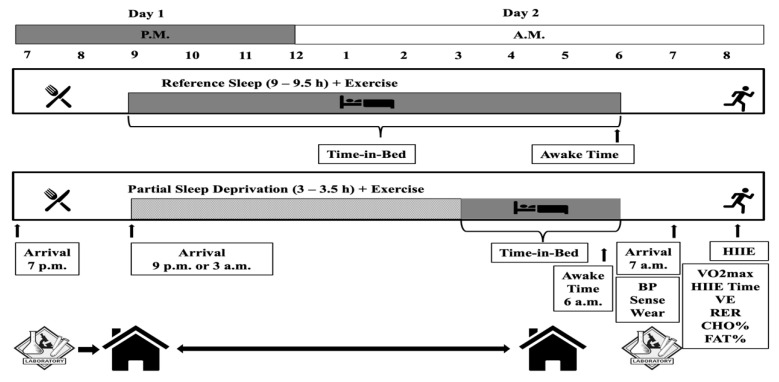
Each experimental condition was completed on two consecutive days and began after 48 h of physical inactivity, no medication use, and the consumption of a diet standardized to what the individual consumed during the first intervention and free from supplementation of any kind. Each experimental condition involved one pre-sleep standard meal consumed in the evening of the first experimental day before the sleep and exercise interventions. The experimental conditions included: (1) A “reference sleep and exercise condition” (RSE) in which a session of high-intensity interval exercise (3:2 intervals at 90 and 40% of VO_2reserve_ that average 70% of VO_2reserve_) that expends 500 kcals of energy was performed after 9–9.5 h of sleep, and (2) a “partial-deprived sleep and exercise condition” (SSE) after 3 to 3.5 h of rest limited to no more than 3 h of sleep in which a session of high-intensity interval exercise expends 500 kcals of energy. Participants arrived at 7 p.m. at the laboratory and stayed until around 8:30 p.m. until they consumed the evening meal. We discharged participants from the lab accounting for the commuting and bed-preparation time, therefore, at 9 p.m. or 3 a.m. all the participants were to be in bed. The participants stayed in their homes until awake time which was set at 6 a.m. During this time, only data from SenseWear were collected to verify the sleep duration. The participants had to be at the lab at 7 a.m. the next day. Between 7 a.m. and 8 a.m., we examined the data collected from the sleep monitor and the pre-exercise resting blood pressure was obtained. After that, an HIIE protocol was executed collecting respiratory gases and obtaining the related variables of interest.

This study was a subpart of a project that examined acute-partial sleep deprivation and cardiometabolic/autonomic nervous responses to HIIE [4,5]. A within-subject randomized crossover experimental design with two experimental conditions was employed. Each experimental condition was completed in a serial manner, with measurements performed the evening of day 1 and the next morning of day 2 (see Figure 1).

Conditions began 48 h after limiting physical activity to activities of daily living, no medication use, and the consumption of a diet standardized to what the individual consumed during the first time of the experimental condition and was free of supplementation of any kind. At least a 72 h washout period occurred between the completion of the experimental condition and initiating the next condition and no more than 2 weeks between the experimental conditions [60].

Experimental conditions included: (1) A RSE in which a meal was ingested in the evening before the reference sleep at day 1 (9 to 9.5 h of time-in-bed in with ~8 h of sleep was attained) followed on day 2 by a session of HIIE (3:2 min intervals at 90 and 40% of VO_2reserve_ that average 70% of VO_2reserve_) that expends 500 kcals of energy, and (2) a SSE similar to RSE but with 3 to 3.5 h of time-in-bed limited to no more than 3.5 h of sleep (see Figure 1). All the participants were asked to read and sign an informed consent form before they took part in the study. The study was conducted in accordance with the Declaration of Helsinki, and the protocol was approved by the Institutional Review Board (#758508-7).

### 2.2. Participants

Eligibility for participation was granted to males, age between 24 and 55, apparently healthy assessed by a modified health and history questionnaire [61], with normal and overweight body mass index (BMI) (18.5–29.9 kg/m^2^). The cohort had to be recreationally physically active engaging in a regular leisure-time or work-related physical activity, but not engaged in training for long-distance endurance events. They had to be non-smokers and not taking any medications known to influence neural and/or cardiometabolic responses. Their respective quality of habitual sleep had to be “good” as indicated by the PSQI of ≤5 [62] and by wearing a sleep monitor [63] for a week long in order to characterize their habitual sleep duration (i.e., ~8 h) [64,65,66,67].

### 2.3. Preliminary Experimental Procedures

#### 2.3.1. Body Composition and Cardiorespiratory Fitness

Before undergoing experimental procedures, the participants’ BF% was determined by dual-energy X-ray absorptiometry (DXA) (Discovery DXA^™^, Hologic^®^, Bedford, MA, USA). After that, we determined their CRF with an individualized treadmill maximal graded exercise test (VO_2max_), as previously described [4,5]. Briefly, for warmup the participants had to jog/walk for 5 min at a self-selected pace. After the warmup, the participants ran in speeds that were increased every 3 min for the first three stages with the last stage representing the participants’ 5 K race pace. The grade was set at 0% for the first three stages and then it was increased by 2% every minute with the speed to be the same as the last 3-min stage. The test was terminated when the participants reached volitional fatigue and were required to stop. Respiratory gases (VO_2_ and VCO_2_) were measured continuously using an integrated respiratory gas analysis system (ParvoMedics, Sandy, UT, USA). We used the results of the CRF to describe their fitness and calculate the respective HIIE intensities for the experimental conditions (see Figure 1) [68].

#### 2.3.2. Sleep, Physical Activity, and Diet

In order to ensure that the participants were “good sleepers” and in addition to the scores of PSQI, a sleep monitor (SenseWear™, Body Media^®^, Pittsburgh, PA, USA) was provided to be worn on their non-dominant arm for 23 h a day for one whole week prior to the experimental conditions, as well as 2 days leading up to the RSE and SSE conditions. Data from the sleep monitor were used to characterize sleep and physical activity levels [63,64,65,66,67,69,70], as the participants had to be “good responders” and to refrain from physical activities for 2 days prior to the RSE and SSE conditions. In addition to sleep and physical activity requirements, the participants had to control and monitor their diets for the same period (i.e., 1 week prior and 2 days leading to the experimental conditions). The participants were instructed to follow their typical diet so they could be easily reproduced for the RSE and SSE conditions. The dietary intake and macronutrient composition were processed using ChooseMyPlate^®^ (US Department of Agriculture, Washington, DC, USA).

### 2.4. Experimental Conditions

#### 2.4.1. Evening Meal

In order to simulate a real-life scenario and avoid negative influences on sleep quality due to the meal composition [42], the participants received a typical light evening meal (non-standardized per body mass) of approximately 805 kcals high and 127 g in carbohydrates (70.4%), fat 20 g (11.1%), and protein 33.3 g (18.5%) macronutrients. The meal was made up of a turkey and cheese sandwich on whole grain bread, a medium banana, a 150 g cup of Greek yogurt, and a 24 oz Gatorade drink. The participants did not have anything else ingested until the experimental conditions the following morning. After finishing their meal, they had to remain in the lab until it was time for them to return to their residence and go to sleep.

#### 2.4.2. Sleep

To avoid any possible anxiety and disruption from occurring during sleeping in an unfamiliar place, participants completed all sleep at their residence. Sleep conditions were designed based on the national recommendations for adults of obtaining 7 to 9 h of sleep [1,71,72]. For the RSE condition, the reference sleep allowed for 9.5 h of bed rest hoping that the participants would have achieved ≥8 h of sleep. For the SSE condition, the acute-partial sleep deprivation allowed for 3.5 h of bed rest and limited sleep to ≤3 h of sleep. Participants had to go straight to their residence once they left the lab. The research design allowed enough time for commuting and preparation time so that the requisite sleep could be obtained for each condition. Upon arriving at their place of residence, we asked them to refrain from eating, watching television, and engaging in computer activities preceding their sleep preparations. We instructed them to both record the time they entered the bed and the wake-up time. The next day’s lab appointment was scheduled to allow enough time for them to prepare and commute to the lab. Participants wore the SenseWear to monitor their sleep duration so the researchers are able to verify whether the participants have met the sleep duration-related inclusion criteria for the RSE and SSE conditions.

#### 2.4.3. High-Intensity Interval Exercise

Each HIIE session was performed under constant laboratory environmental conditions on a motorized treadmill Trackmaster^®^ TMX 428CP^™^ and began at least 10–12 h after the evening meal in a fasted condition at about 0800 h using a specific HIIE protocol, as previously described [4,5,71,72,73,74,75,76,77]. At first, a resting blood pressure was obtained following the standard procedures and a mean arterial pressure was calculated [68] followed by the HIIE. The HIIE protocol included a brief 5-min warm-up period consisting of walking at 2.5 mph and 0% grade. The HIIE session was completed in 3-min running intervals at 90% of VO_2reserve_ separated by 2-min intervals of jogging/walking at 40% of VO_2reserve_. The average intensity of all HIIE sessions was equated to 70% of VO_2reserve_. The HIIE session was concluded when the participants expended a total of 500 kcal, as determined by the continuous respiratory gas analysis. Respiratory variables of volume of oxygen (VO_2_), volume of carbon dioxide (VCO_2_), expired ventilation (VE), respiratory exchange ratio (RER), etc. were measured continuously using an integrated respiratory gas analysis system after a standardized calibration before each measurement.

### 2.5. Statistical Analyses

Dependent variables included the relative VO_2max_, time needed to expend 500 kcal, VE, RER, carbohydrate percent (CHO%) utilized, fat percent (FAT%) utilized, and resting mean arterial pressure (MAP) of the morning of day 2 before the HIIE. Independent variables included the experimental conditions, RSE and SSE (see Figure 1). Descriptive characteristics are presented in Table 1. Substrates utilization for CHO% and FAT% were calculated based on the concept that lipid oxidation is related to RER values < 0.71, whereas a value of RER > 1.00 is related to the CHO oxidation. Considering that RER values greater than 0.85 represent an index of fat to the CHO oxidation assuming that protein oxidation was negligible throughout the HIIE, the respective percentages were calculated using the following formulas [78]:

(1.00 − RER)/(1.00 − 0.70) × 100 = %FAT utilized

100% − %FAT = %CHO utilized,

The Kolmogorov-Smirnov/Shapiro-Wilk and normal Q-Q plots were used to determine the normality of the data [79]. In the case of violation of normality, natural logarithmic transformations were performed. In the case of violation of sphericity, the Greenhouse-Geisser degrees-of-freedom correction was performed.

Since this study was a subpart of a bigger project with different dependent variables that required at least a sample size of 15 participants [80], a separate power analysis was run to verify if such a sample size was adequate enough to answer the research hypothesis of this study. Previous studies related to research questions similar to this project used seven to 14 participants [6,10,14,18,22,23,24,81,82,83,84,85,86]. Moreover, using the G*Power for Mac (vs. 3.1.94, 2009) for the F-test, ANOVA repeated measures, within the interaction with the alpha level at 0.05, power at 0.80, one group, lower bound of sphericity at 1, and effect size at 0.4, yielded a total sample size of 12 [87]. Therefore, a sample of 15 participants was considered proper to infer statistical significance and extrapolate the results for the aims of this study.

Data were analyzed using the repeated measures ANOVA for conditions (RSE vs. SSE) to examine differences in the main variables of interests. Moreover, an ANCOVA with VO_2max_ and BF% as covariates was run to compare the mean response of the dependent variables. The Bonferroni post-hoc procedure was used to follow-up significant findings. Eta squared (η^2^) effects sizes were also calculated with the following threshold values: <0.2 trivial, >0.2 small, >0.6 moderate, and >1.2 large [88]. The statistical significance for this study was set *a priori* with a *p*-value ≤ 0.05. Group characteristics were reported as the mean ± SD and data analyses were completed with the IBM Statistical Package for Social Sciences (SPSS) for Mac, v. 26, (IBM Corp., Armonk, NY, USA).

## 3. Results

### 3.1. Pre-Experimental Data

#### Caloric Intake, Physical Activity, and Sleep

A total of 30 individuals met the inclusion criteria, but only 15 were able to adhere to the study’s protocol, complete the study, and be included in the statistical analysis. Descriptive data for the 7 days and baseline are presented in Table 1.

The selected cohort included habitually good sleepers which are able to follow the study’s aims, as determined from the sleep data. Participants did not differ in their sleep patterns during the 7 days with respect to the sleep laying down time (F_3.8,52.8_ = 0.41, *p* = 0.79, η^2^ = 0.028), sleep duration (F_3.5,49.2_ = 0.48, *p* = 0.73, η^2^ = 0.033), and sleep efficiency (F_3.1,43.3_ = 0.92, *p* = 0.44, η^2^ = 0.062). Moreover, participants’ sleep laying down time (F_1,14_ = 0.85, *p* = 0.37, η^2^ = 0.057), sleep duration (F_1,14_ = 0.37, *p* = 0.55, η^2^ = 0.026), and sleep efficiency (F_1,14_ = 3.7, *p* = 0.08, η^2^ = 0.210) did not differ during the 7 days of data collection and RSE.

### 3.2. Experimental Data

#### 3.2.1. Caloric Intake, Physical Activity, and Sleep

The cohort’s caloric intake did not differ between the 7 days, RSE, and SSE (F_2,28_ = 2.22, *p* = 0.13, η^2^ = 0.137). Physical activity levels did not differ between the 7 days, RSE, and SSE (F_2,28_ = 2.34, *p* = 0.12, η^2^ = 0.143) and there was no difference in the antioxidant consumption between the 7 days, RSE, SSE in vitamin A (F_1.39,19.4_ = 2.04, *p* = 0.15, η^2^ = 0.127), vitamin C (F_2,28_ = 0.24, *p* = 0.79, η^2^ = 0.017), and vitamin E (F_2,28_ = 1.4, *p* = 0.27, η^2^ = 0.090).

Data for RSE and RSE regarding diet, physical activity, and sleep are presented in Table 2. The caloric intake did not differ between RSE and SSE (F_1,14_ = 0.36, *p* = 0.56, η^2^ = 0.025). Physical activity levels did not differ between RSE and SSE (F_1,14_ = 2.73, *p* = 0.12, η^2^ = 0.163). Vitamin A (F_1,14_ = 0.01, *p* = 0.94, η^2^ = 0.006), vitamin C (F_1,14_ = 0.07, *p* = 0.80, η^2^ = 0.006), and vitamin E (F_1,14_ = 0.60, *p* = 0.45, η^2^ = 0.041) did not differ between RSE and SSE. By design, there was a difference in the sleep laying down (F_1,14_ = 297.98, *p* < 0.01, η^2^ = 0.955), sleep duration (F_1,14_ = 285.67, *p* < 0.01, η^2^ = 0.953) between RSE and SSE, but not in sleep efficiency (F_1,14_ = 2.07, *p* = 0.17, η^2^ = 0.129).

#### 3.2.2. Exercise and Substrate Utilization

Descriptive data for exercise and substrate utilization are presented in Table 3. A covariate analysis with VO_2max_ and BF% on the main variables of interest did not alter the outcomes, therefore we present results only for the repeated ANOVA. In general, no differences between RSE and SSE were observed in any of the examined HIIE performance variables, but there was a possible trend of an impaired HIIE aerobic performance. No difference between RSE and SSE in substrate utilization during HIIE was observed as well, but during the RSE more CHO (89%) was used compared to RSE (86%), while fat was utilized less at RSE (11%) compared to SSE (14%). A significant difference in MAP was reported between conditions with participants presenting a lower resting MAP before the HIIE at the SSE compared to RSE.

## 4. Discussion

This study attempted to explore the impact of acute-partial sleep deprivation on the next day’s HIIE performance and subsequent substrate utilization after an overnight fasting. We hypothesized that the HIIE performance in the sleep deprived condition will be impaired, but results presented that the HIIE performance was not influenced by acute-partial sleep deprivation, even though a trend for a marginal decrease emerged. Moreover, we hypothesized that participants in the sleep deprived condition will be using more CHO% and less FAT% during the HIIE, but on the contrary, results showed that partial sleep deprived participants utilized more FAT% and less CHO% compared to the reference sleep and HIIE. In addition, our cohort presented at the morning of the second day of the sleep deprived condition with lower resting mean arterial pressure, as recorded before the HIIE. When we added in the examined model participants’ respective VO_2max_ and BF%, there was still no difference in the earlier reported outcomes.

It is difficult to position the current findings in the context of the sleep and aerobic performance due to the documented discrepancies on selected populations, durations of sleep deprivation, and the time of day that was induced, and lastly the performance assessment protocols [19,26]. Our results support the current ambiguity surrounding the studies that examine partial sleep and aerobic performance by employing sub-maximal or maximal protocols [28].

For example, the morning (8 a.m.) cycling performance was impaired at the partial sleep deprived condition (~2.5 h) compared to the reference sleep (~7 h) for six male and two female recreational cyclists [6]. Chase et al. reported a 4% decrease in time completion of 3-km cycling after sleep restriction, and in this context, our findings are in direct disagreement as we did not observe any difference, only a minimal impairment in time performance for the SSE. Chase et al. even though they reported changes in time performance, similar to us, they did not report any difference at VO_2_, VE, and RER suggesting that these variables probably are not influenced by sleep deprivation [6,28,89], even at extreme levels of 64 h of deprivation [90].

There is, however, some evidence supporting the fact that partial sleep deprivation can alter heart rate, VE, and RER due to the elevated catecholamine and sympathetic nervous system for exercise intensities over 75% of VO_2max_ [23,26,91]. We did not measure heart rates, but our cohort presented in the sleep deprived condition with significantly lower mean arterial pressure indicating that they possibly had lowered heart rates and their sympathetic nervous system was not stressed enough, and the parasympathetic branch was more activated. This can also be supported by the heart-rate variability (HRV) indices related to this study, which were recently presented from the same project. We reported that acute-partial sleep deprivation significantly increased HRV indices, such as high-frequency (HF) and root mean square of successive normal RR interval differences (RMSSD), which are mainly influenced by the parasympathetic activation in the morning after the sleep deprivation on day 2 before the HIIE [5]. There is a lack of studies examining acute-partial sleep deprivation and HRV, but it has been reported that partial deprivation increases the sympathetic branch of the autonomic nervous system as reflected by an increase in low frequency (LF) and decrease in HF [92].

The evening cycling exercise at 75% of the VO_2max_ for at least 20 min after partial sleep deprivation of 3 h was able to significantly reduce the aerobic performance VO_2max_, increase both VE and lactate accumulation compared to ~8.5 h of reference sleep on trained endurance male athletes, but no significant difference in the maximal exercise intensity was reported [23]. Their exercise intensity was averaged to 75% of the predetermined maximal oxygen consumption, but exercise was performed during the afternoon hours on a cycle ergometer. Previous work has shown that heart rate, VE, and RER during exercise are not impacted by sleep patterns [28,89]. However, there is some support that sleep deprivation can influence physiological variables when exercise is performed at relatively high intensities greater than 75% of VO_2max_ due to the induced stress on the cardiovascular system and elevation of the catecholamine levels [23]. In our study, we utilized a similar average exercise intensity (70% of VO_2max_), but it was performed during the morning hours in a HIIE form and on a treadmill where the sleep debt was not yet high enough. It has been reported that the morning performance is unaffected by the sleep patterns of the previous night and this may be the reason for the partial discrepancy in the findings between our study and Mougin et al., while the evening performance is more likely to be affected as the sleep debt accumulates [13,14,19,93].

Cullen et al. reported that a 15-min all out cycling aerobic performance was significantly decreased in the next morning (7 to 9 a.m.) in recreationally active males after 4 h of sleep compared to the reference sleep [10]. Similarly, trained cyclists/triathletes also reported an impaired cycling morning endurance time-trial for more than 50 min of performance in the sleep deprived condition (~4.5 h) compared to the reference sleep (~7 h) [25]. In addition to the apparent similarities and differences in the design of the previous two studies and ours, a possible reason for the discrepancy between their results and ours (i.e., no difference in the HIIE performance) may be due to the discomfort which is associated with all our tests and the long endurance tests compared to the submaximal workouts as ours. It is possible that the applied HIIE protocol during the SSE was perceived in a more pleasant way eliciting less discomfort and allowing our participants to exercise at the same levels as the RSE [94].

The results of this work agree with Omiya et al. who reported that healthy men presented a non-significant decrement in the cycling cardiopulmonary exercise test evening performance (5–9 p.m.) after a 3 h sleep deprivation compared to the control sleep of ~7.5 h. They also reported that time to exhaustion, peak heart rate, and maximum oxygen consumption was not affected by sleep loss [20]. In addition, our results are partially confirmed by Mougin et al. who reported no difference in the maximal cycling exercise intensity. However, their evening cycling exercise at 75% of the VO_2max_ for at least 20 min after partial sleep deprivation of 3 h was able to significantly reduce the aerobic performance VO_2max_, increase both VE and lactate accumulation compared to ~8.5 h of reference sleep on trained endurance male cyclists [23].

Mejri et al. reported no change in the intermittent aerobic performance test scores on Taekwondo players at 7–8 a.m. after partial sleep deprivation of 3 h compared to 7.5 h of reference sleep [19]. Our findings agree with Mejri et al. and even though their sample was Judokas, while ours was recreationally active people, based on the Judokas respective CRF and BF% levels many of our participants could have been categorized as athletes, as well. Having high fitness levels has been associated with better sleep quality and adopting a healthy lifestyle [3] may have masked our cohort’s responses to sleep deprivation due to their respective fitness (VO_2max_-49.2 mL/kg/min) and due to the research design, that recruited recreationally active, normal, and overweight non-smokers individuals. It is possible that if participants were not that fit and following a sedentary lifestyle to have different HIIE performance and metabolic responses with respect to overnight-fasted partial sleep deprivation. Moreover, our study and Mejri’s et al. study had similar sleep deprivation (~3 h) and reference sleep (~7.5 h) protocols, exercise was performed in the morning and involved running as the mode of exercise. In addition, even though there is a notable difference between submaximal and maximal tests performed in the laboratory versus in the field or on a treadmill versus on a cycling ergometer, both applied exercise stimuli probably stressed the same metabolic pathways. The Yo-Yo test evaluates the capacity to perform repeated high intensity aerobic work with the anerobic system to be taxed towards the end of the test [95,96], similar to our HIIE protocol as demonstrated by the respective RER values.

The fasted exercise at about 70% of VO_2max_ mobilizes and promotes fat oxidation via increased lipolytic activity and limited action of insulin and in that respect our findings support the literature [55]. The energy substrate during exercise depends on the intensity, duration, and level of training and FAT% utilization is higher at no greater than 60–65% of VO_2max_ and decreases at intensities greater than 75% of VO_2max_. Even though the applied HIIE protocol involved intensities at 90% of VO_2max_ for 3 min and 40% of VO_2max_ for 2 min the average interval intensity was 70% of VO_2max_ indicating that the observed increase in FAT% during the acute-partial sleep deprivation is justified [55]. The nutrient substrate utilization and most notable the muscle glycogen storage and utilization is impaired, especially during sleep deprivation where the brain is “hungry” for glucose [97,98]. It has been shown that sleep deprivation of 30 h reduces muscle glycogen and affects brain and glucose metabolism [99,100]. In five and a half hours of a sleep deprivation protocol, CHO% is augmented due to the increased need of glucose in the brain metabolism during sleep deprivation. Results from the same study reported increased hunger and elevated fasting and postprandial respiratory quotient (RQ) values with reduced FAT% oxidation and with no increase in catabolic hormones such as cortisol, triiodothyronine, free thyroxine, and catecholamines [61]. We did not deprive our participants nor did we measure muscle glycogen, ghrelin, leptin or catabolic hormones, but based on the slightly lower obtained RER values from the partial sleep deprived condition and the respective decrease in CHO% and increase FAT% utilization during the HIIE, we may suggest that the same levels of oxidative glucose metabolism occurred, with no noticeable impairment in glucose metabolism. Taking all these together is premature to conclude on whether partitioning of the substrate utilization was affected by the acute-partial sleep deprivation. In theory, the extra time spent, while being awake induces an increased demand for glucose to support brain wakefulness and other glucose dependent tissues while sparing fat [62].

An increase in the exercise intensity and/or duration alters the cardiac autonomic modulation with sleep quality to be independent of intensity and duration as aerobic exercise is not able to result in sleep pattern disturbances [101]. In that context, many studies do not report that sleep related characteristics add more in the complexity of interpreting the literature. In our study, sleep efficiency for the reference sleep was 86%, with the minimum efficiency recommended for good health [102] to be 85%, and for the partial sleep deprived condition to be 81%. Moreover, it has been shown that the timing of the food intake and its composition are correlated with negative effects on sleep quality [43]. Our study’s design with its light meal composition allowed for adequate time between the last meal and preparations for sleep, while supported the body with CHO to improve sleep and the next day’s aerobic performance [103]. It is apparent that all of the aforementioned studies, including ours, have used different populations, applied exercise protocols, and patterns of sleep manipulation, factors that have been discussed to be reasons for discrepancies [19,26].

The present study has several strengths. First, 15 participants were used for the statistical analysis when the common sample size is between 7 and 14 providing adequate statistical power when testing the null hypothesis [104]. However, recently it was suggested that the Bayesian analysis may be a more appropriate statistical analysis to detect subtle changes in the partial sleep deprivation area overcoming the common problems of large variability and low sample sizes [10]. Studies may consider examining their research questions not only using inferential statistics, but also under the Bayesian theorem, recognizing that even this approach has its limitations, as well [105]. However, given the crossover nature of the study and the power size calculations, this is unlikely to have affected the findings. In an effort to eliminate the common trait in variability in individual responses among studies regarding acute-partial sleep deprivation, we utilized a strict diet and sleep protocol for 7 days to verify eligibility and again 2 days prior to the experimental conditions to ensure that the sample would be “habitually good sleepers” and to respond adequately to acute-partial sleep deprivation [89,106]. In addition, using a treadmill rather than a cycling ergometer and HIIE protocol of that nature was something unique that we have not experienced before in the literature. Moreover, the majority of studies examining sleep loss acute or partial on performance lack specificity as their “scenarios” are not applicable to real life [26]. This study’s protocol has a real life’s applicability and significance, as exercising early in the morning after an overnight fasting and acute sleep deprivation is a common behavior [50,51].

However, this study is limited in employing objective sleep quality measurements that assess sleep architecture and cycles. We defined sleep patterns as having subjective evaluations based on PSQI scores and objective using the sleep monitor. Such measures have proven to have good applicability, reproducibility, face validity, and they are relatively inexpensive in nature [65,66,68,72,107].

It is important to consider that our findings are specific to the time of day (7:00–8:00 a.m.), on how awake or restored the participants were from the previous night in questioning or even by their respective chronotype (i.e., morning-type or evening-type) [108,109,110]. The time of day has been shown to influence the level of aerobic performance such as time to exhaustion and total distance covered in the Yo-Yo test [111,112]. The time of day may have an effect on the performance of the muscle contractile properties and the intracellular variation due to the circadian rhythm impact on the inorganic phosphate concentration and central temperature which would influence calcium release from the sarcoplasmic reticulum [113]. It has been shown that morning-type individuals perform better in the morning, while evening-type individuals have their peak performance in the evening during a self-paced walking task [114]. It is reported that evening-type individuals will meet more of a burden when they perform a HIIE protocol (e.g., 4 × 4 min at 90–95% of peak heart rate with 3 min of active recovery at 50–60% of peak heart rate) early in the day [115]. Since we did not assess the participants’ circadian typology, we are not in a position to determine if our results were attributed due to the sample’s distribution of morning-type and evening-type participants and how these responded to an 8:00 a.m. HIIE session.

Restrictions can be imposed due to the specific design and studied population highlighting the need for larger studies performed in sleep research centers with other population groups, such as women and sedentary individuals, as well as studies that share similar or different HIIE protocols and modes of exercise. A per design exercise was scheduled in the morning, and we instructed the participants to avoid strenuous physical activity for at least 48 h prior to the sleep intervention. This practice may have masked our reported acute-partial sleep deprivation and next-day HIIE performance responses due to the given restorative nature of sleep on regulating homeostasis [35,102,116]. Moreover, comparing morning and evening exercisers and/or gender responses under the partial sleep deprived condition is still unexplored. In sleep related studies, there is very often an issue with the operational definition of what “short” and “long” sleep loss are across different studies, thus preventing adequate comparisons [18].

Another issue that may complicate the interpretation of this study’s results may be the heterogeneity of the included covariates. Many have included age and gender, health status and history, sociodemographic and socioeconomic factors, and medication use [18]. Therefore, future studies may consider the inclusion of more covariates, since not adjusting for enough variables may lead to relationships and differences caused by several factors, other than the casual effects of sleep.

Future studies may examine this research question using different HIIE protocols/modalities and establish a more solid proof with regards to the effect of sleep patterns (duration and quality) in the overnight-fasted HIIE performance and metabolism. More research is needed to confirm acute sleep deprivation effects on intermittent bouts of exercise, maybe on sport specific and cognitive performance. It is critical to obtain a better understanding on the physiological/psychosocial interactions of acute-partial sleep and exercise, glucose tolerance, obesity, and cardiovascular functioning. In addition, studying sleep disturbance in accordance to sleep duration, quality, and latency will provide a better understanding of the implications of short sleep for the public health.

## 5. Conclusions

Our findings suggest that acute-partial sleep deprivation has no impact on the HIIE performance and utilizes less CHO% and more FAT% during HIIE in the morning hours. In that perspective, HIIE in the fasted acute-partial sleep deprived state provides a neglectable increase in fat oxidation compared to the reference sleep HIIE. Therefore, it is probably wiser for people that practice the HIIE session in the morning hours to perform as such under a full night of sleep, in order to receive the sleeps’ pleiotropic beneficial effects. Moreover, exercise specialists and health care related practitioners may find this information useful when they prescribe exercise for health and well-being.

## Figures and Tables

**Table 1:** Baseline screening −7 days of diet, activity levels, and sleep.

Variable	Mean ± SD	Min	Max
Age (year)	31.1 ± 5.3	24	40
Height (cm)	179.3 ± 6.8	167.6	188.0
Weight (kg)	83.3 ± 11.4	70.7	105.7
BMI (kg/m^2^)	25.8 ± 2.7	21.1	29.9
BF (%)	21.0 ± 6.5	11.4	35.3
VO_2_ (mL/kg/min)	49.2 ± 8.5	36.0	66.0
Time to complete VO_2_ test (min)	10.1 ± 1.9	6.5	13.4
VE (L/min)	68.9 ± 17.3	32	95
RER	0.97 ± 0.5	0.89	1.03
PSQI	3.7 ± 0.9	2	5
7 Days Caloric intake (kcal)	2372 ± 576	1421	3440
7 Days Activity levels (METs)	1.49 ± 0.15	1.3	1.7
7 Days SL (h:mm:ss)	8:25:44 ± 1:22:50	6:33:00	11:15:00
7 Days SD (h:mm:ss)	6:43:28 ± 1:22:50	5:03:00	9:11:00
7 Days SLE (%)	81 ± 11	55.19	91.1

All values are presented as mean ± standard deviation. VO_2_: Volume of oxygen consumption; VE: Expired ventilation; RER: Respiratory exchange ratio; BF: Body fat; BMI: Body mass index; PSQI: Pittsburgh sleep quality index; SL: Sleep plus laying down; SD: Sleep duration; SLE: Sleep efficiency expressed as the percentage of sleep duration over the laying down time; METs: Metabolic equivalents; activity levels are labeled as sedentary (up to 1.5 METs), light (1.5–3.0 METs), and moderate (3.0–6.0 METs).

**Table 2:** Sleep and physical activity level conditions.

Variable	RSE	SSE
Caloric intake (kcal)	1989 ± 749	2106 ± 870
Activity levels (METs)	1.4 ± 0.15	1.5 ± 0.15
Vitamin A (μg)	782.53 ± 618.17	795 ± 603.30
Vitamin C (μg)	156.73 ± 378.91	191.1 ± 376.41
Vitamin E (μg)	11.8 ± 7.86	14.53 ± 12.62
SL (h:mm:ss)	8:11:04 ± 1:01:48	3:18:09 ± 0:52:02 *; *p* < 0.05
SD (h:mm:ss)	6:57:09 ± 0:47:38	2:39:30 ± 0:40:11 *; *p* < 0.05
SLE (%)	86 ± 8	81 ± 12

All values are presented as mean ± standard deviation. Means with * are significantly different between conditions at *p* < 0.05. SL: Sleep plus laying down; SD: Sleep duration; SLE: Sleep efficiency expressed as the percentage of sleep duration over the laying down time; activity levels are labeled as sedentary (up to 1.5 METs), light (1.5–3.0 METs), and moderate (3.0–6.0 METs); METs: Metabolic equivalents; RSE: Reference sleep and high-intensity exercise; SSE: Acute-partial sleep deprivation and high-intensity interval exercise.

**Table 3:** Resting mean arterial pressure, exercise performance, and substrate utilization.

Variable	RSE	SSE	Within-Subjects Effects
90% VO_2reserve_ (mL/kg/min)	41.6 ± 7.3	41.2 ± 7.3	F_1,14_ = 0.30, *p* = 0.6, η^2^ = 0.021
40% VO_2reserve_ (mL/kg/min)	20.4 ± 3.2	20.3 ± 3.1	F_1,14_ = 0.03, *p* = 0.87, η^2^ = 0.002
VO_2max_ (mL/kg/min)	45.8 ± 8.1	45.39 ± 8.1	F_1,14_ = 0.22, *p* = 0.6, η^2^ = 0.016
Time to complete VO_2_ test (min)	24.31 ± 2.6	24.44 ± 2	F_1,14_ = 0.09, *p* = 0.77, η^2^ = 0.006
VE (L/min)	72.8 ± 9.39	71.6 ± 10.84	F_1,14_ = 0.41, *p* = 0.53, η^2^ = 0.029
RER	0.97 ± 0.037	0.96 ± 0.041	F_1,14_ = 0.41, *p* = 0.53, η^2^ = 0.029
CHO (%)	88.91 ± 12.63	86.65 ± 13.94	F_1,14_ = 0.41, *p* = 0.53, η^2^ = 0.029
FAT (%)	11.08 ± 12.63	13.34 ± 13.94	F_1,14_ = 0.41, *p* = 0.53, η^2^ = 0.029
MAP (mmHg)	98.6 ± 6.3	72 ± 9.8	F_1,14_ = 222.97, *p* < 0.001, η^2^ = 0.941 *

All values are presented as mean ± standard deviation. Means with * are significantly different between conditions. VO_2max_: Maximum volume of oxygen consumption; VE: Expired ventilation; RER: Respiratory exchange ratio; CHO: Carbohydrate percentage utilized; FAT: Fat percentage utilized; MAP: Resting mean arterial pressure; RSE: Reference sleep and high-intensity exercise; SSE: Acute-partial sleep deprivation and high-intensity interval exercise.

## Data Availability

The datasets generated during and/or analyzed during the current study are available from the corresponding author on a reasonable request.
